# Promising Effects of Neurorestorative Diets on Motor, Cognitive, and Gastrointestinal Dysfunction after Symptom Development in a Mouse Model of Parkinson's Disease

**DOI:** 10.3389/fnagi.2017.00057

**Published:** 2017-03-20

**Authors:** Paula Perez-Pardo, Esther M. de Jong, Laus M. Broersen, Nick van Wijk, Amos Attali, Johan Garssen, Aletta D. Kraneveld

**Affiliations:** ^1^Division of Pharmacology, Utrecht Institute for Pharmaceutical Sciences, Faculty of Science, Utrecht UniversityUtrecht, Netherlands; ^2^Nutricia ResearchUtrecht, Netherlands

**Keywords:** Rotenone Parkinson's model, motor-symptoms, non-motor symptoms, uridine, docosahexaenoic acid

## Abstract

Parkinson's disease (PD) is characterized by the progressive degeneration of dopaminergic nigrostriatal neurons, with reductions in the function and amount of dopaminergic synapses. Therefore, synapse loss and membrane-related pathology provide relevant targets for interventions in PD. We previously showed the beneficial preventive effects of a dietary intervention containing uridine and DHA, two precursors for membrane synthesis, in the intrastriatal rotenone model for PD. Here, we examined the therapeutic potential of the same dietary intervention on motor, cognitive, and gastrointestinal symptoms. In addition, we tested the effects of an extended nutritional formula based on the same precursors plus other nutrients that increase membrane phospholipid synthesis as well as prebiotic fibers. C57BL/6J mice received a unilateral rotenone injection in the striatum. Dietary interventions started 28 days after surgery, when motor-symptoms had developed. Readout parameters included behavioral tasks measuring motor function and spatial memory as well as intestinal function and histological examination of brain and gut to assess PD-like pathology. Our results show that rotenone-induced motor and non-motor problems were partially alleviated by the therapeutic dietary interventions providing uridine and DHA. The extended nutritional intervention containing both precursors and other nutrients that increase phospholipid synthesis as well as prebiotic fibers was more effective in normalizing rotenone-induced motor and non-motor abnormalities. The latter diet also restored striatal DAT levels, indicating its neurorestorative properties. This is the first study demonstrating beneficial effects of specific dietary interventions, given after full development of symptoms, on a broad spectrum of motor and non-motor symptoms in a mouse model for PD.

## Introduction

Parkinson's disease (PD) is the second most common neurodegenerative disease after Alzheimer's disease (AD; de Rijk et al., 2000; Nussbaum and Ellis, 2003). The clinical picture is dominated by motor impairments due to a progressive degeneration of dopaminergic nigrostriatal neurons, with reductions in striatal dopamine levels, dopaminergic synapses, and the density of dendritic spines on striatal medium spiny neurons (Albin et al., 1989; Crossman, 1989). PD patients also develop non-motor symptoms, including cognitive impairment (Aarsland et al., 2003) and gastrointestinal (GI) dysfunctions (Pfeiffer, 2011; Fasano et al., 2015). These non-motor symptoms are major determinants of quality of life (Schrag et al., 2000; Martinez-Martin et al., 2011; Müller et al., 2013) and remain undertreated (Chaudhuri and Schapira, 2009).

The most commonly used drug in the treatment of PD is L-Dopa that compensates for dopaminergic cell loss by enhancing dopamine synthesis in the remaining terminals. This therapy has several side effects (Schrag and Quinn, 2000), it does not prevent dopaminergic neuron degeneration, and has no effects on non-motor symptoms (Lee and Koh, 2015). Considering that some of the non-responsive symptoms like GI dysfunctions may contribute to L-Dopa response fluctuations (Poewe et al., 2010), there is a clear need to develop additional therapies for PD treatment.

Oral administration of two circulating phosphatide precursors, uridine, and docosahexaenoic acid (DHA), increases dopaminergic neurotransmission, synaptic membrane formation, as well as the density of dendritic spines (Wang et al., 2005; Sakamoto et al., 2007; Wurtman et al., 2009, 2010). Indeed, preventive treatment with precursors uridine and DHA reduced rotational behavior in the unilateral 6-OHDA rat model for PD (Cansev et al., 2008). We have also shown beneficial preventive effects of a dietary intervention containing uridine and DHA in the intrastriatal rotenone mouse model for PD. Intrastriatal injection of rotenone caused several motor and non-motor symptoms associated with PD. The preventive dietary intervention was not only effective for the motor symptoms but also for the GI phenotype (Perez-Pardo et al., 2017).

In the present study we examine the therapeutic potential of the same dietary intervention in the intrastriatal rotenone mouse model of PD given after the development of full motor symptoms, i.e., 4 weeks after rotenone injection, to elucidate if the diet has neurorestorative properties. We also investigate the therapeutic potential of an extended diet containing the same dietary precursors (uridine and DHA) plus additional nutrients that increase membrane phospholipid synthesis and prebiotic fibers. The additional nutrients displayed synergistic effects with the precursors in enhancing synapse formation and function, in counteracting loss of functional connectivity in neurodegeneration, and in improving behavior and cognitive functions (van Wijk et al., 2014). In mouse models of Alzheimer-like pathology, the extended diet showed higher efficacy than interventions with single nutrients or incomplete formulations (Broersen et al., 2013; Zerbi et al., 2014). Prebiotic fibers have been shown to have beneficial effects on immune function (van Hoffen et al., 2009; de Kivit et al., 2011; Jeurink et al., 2013), bowel motility and constipation (Rasmussen et al., 2014; Scholtens et al., 2014; Meksawan et al., 2016) relevant for inflammation and GI-related symptoms in PD.

## Materials and methods

### Mice

Seven week-old C57BL/6J mice (Charles River, The Netherlands) were housed at room temperature under 12 h light/dark cycle. Food and water was provided *ad libitum*. All animal procedures were approved by the Ethical Committee of Animal Research of Utrecht University, The Netherlands.

### Surgery

Mice underwent stereotaxic surgery under isoflurane anesthesia: a hole was drilled in the skull, a cannula inserted in the right striatum and 5.4 μg of freshly prepared rotenone (dissolved in 2 μl DMSO) was infused. The following stereotaxic coordinates were used: AP +0.4, ML −2.0 (from bregma) and DV −3.3 below dura. Sham-treated animals were injected with vehicle. Ten weeks after surgery the mice were euthanized by decapitation.

### Diets

Mice were fed either the Control diet, Diet1, or Diet2, starting 28 days after surgery when motor symptoms leveled out and continuing for the duration of the experiment. Animals were divided into six groups (*n* = 10), as specified in Table 1. Iso-caloric diets were produced by Research Diet Services (Wijk bij Duurstede, The Netherlands) and were based on the Control diet, i.e., the standard animal food for laboratory rodents AIN-93 M (Reeves et al., 1993) with 5% fat. For Diet1, UMP as a source of uridine (0.51/100 g diet) was added and part of the lipid blend of control diet was replaced by fish oil, providing DHA (0.74/100 g diet) and EPA (0.29/100 g diet).

**Table 1:** **Specification of the 6 experimental groups**.

**Experimental groups**		**Nutritional intervention**		
		**Control diet**	**Diet1**	**Diet2**
Surgery	Sham	1. Sham surgery + Control diet throughout the experiment	2. Sham surgery + Diet1 starting 4 weeks after surgery	3. Sham surgery + Diet2 starting 4 weeks after surgery
	Rotenone	4. Rotenone injection + Control diet throughout the experiment	5. Rotenone injection + Diet1 starting 4 weeks after surgery	6. Rotenone injection + Diet2 starting 4 weeks after surgery

Diet2 provided the phospholipid precursors from Diet1, but also choline, phospholipids, selenium, folic acid, and vitamins B6, B12, C, D, and E. The fish oil in Diet2 provided DHA (0.75/100 g diet) and EPA (0.50/100 g diet). In addition, the cellulose fibers from Control diet and Diet1 were replaced by prebiotic fibers (1.5/100 g diet GOS, 0.17/100 g diet lcFOS, 1.67/100 g diet scFOS, and 1.67/100 g diet nutriose) for Diet2. Further, specifications of the diets are listed in Table 2.

**Table 2:** **Compositions of the experimental diets**.

**Experimental diets**	**Control (g/100 g)**	**Diet 1 (g/100 g)**	**Diet 2 (g/100 g)**
Proteins	14.0	14.0	14.0
Carbohydrates	71.0	70.6	68.7
Fats	5.0	5.0	5.0
Soy oil	1.9		
Coconut oil	0.9	0.1	0.2
Corn oil	2.2	1.7	1.2
Fish oil		3.2	3.6
Providing DHA		0.74	0.75
Providing EPA		0.29	0.50
Mineral mix (AIN-93M-MX)	3.5	3.5	3.5
Vitamin mix (AIN-93-VX)	1.0	1.0	1.0
Fibers	5.0	5.0	5.0
Cellulose	5.0	5.0	
GOS			1.50
IcFOS			0.17
ScFOS			1.67
Nutriose			1.67
Additions			
L-cystine	0.18	0.18	0.18
Choline bitartrate	0.25	0.25	0.25
Tert-butylhydroquinone	0.0008	0.0008	0.0008
Uridine		0.51	0.51
Choline chloride			0.40
Soy lecithin			0.75
Sodium selenite			0.00023
Pyridoxine			0.0041
Folic acid			0.00067
Cyanocobalamin			0.058
Ascorbic acid			0.16
Di-a-tocopheryl acetate (500 IU/g)			0.47
Cholecalciferol (400,000 IU/g)			0.00031

### Motor symptoms assessment

The motor function of each mouse was assessed by the Rotarod test as described before (Inden et al., 2007). Briefly, mice were placed on an accelerating rod with speeds starting with 2 rpm and gradually increasing to 20 rpm. Time to first fall was recorded for a maximum of 300 s. The test was performed at baseline and after every 5 days until day 70, in order to look at symptom development in time and functional recovery during dietary intervention.

Muscle strength of the four limbs was measured on day 70 after surgery using the inverted screen test as described before (Deacon, 2013). Briefly, the mouse was placed in the center of a wire mesh screen, which was subsequently rotated to an inverted position. Latency to fall was measured in seconds.

Muscular forelimb strength was also measured on day 70 with a grip strength tester as described before (Leiter et al., 2011).

### Spatial recognition test

Animals' ability to react to a spatial novelty after a 3-min delay was measured every 14 days after surgery and for the duration of the experiment, to assess model- and diet-induced changes over time. As described before (De Leonibus et al., 2007), mice were individually submitted to seven consecutive, 6-min sessions. During session 1, mice were placed into the empty open field. During sessions 2–4, five objects were present, and mice were placed into the apparatus to habituate to the objects configuration (habituation phase). During the 3-min intersession interval, the animals were returned to a waiting cage. During the spatial test session (session 5), the objects configuration was changed by moving two objects (displaced objects, DO) and leaving the other three objects in the same position (non-displaced objects, NDO). In all sessions, the total activity of the animal was measured. From sessions 2–5, object exploration was evaluated on the basis of the mean time spent by the animal in contact with the different objects. The animals' ability to selectively react to the spatial change was analyzed by calculating the spatial re-exploration index (DO[S5] − DO[S4] = DO and NDO[S5] − NDO[S4] = NDO). The time the animals interact with the DO minus the time they interact with the NDO is used for analysis (DO-NDO). Data are expressed as percentage with respect to baseline (day 14).

### Intestinal transit and colon length

Intestinal transit was assessed in all animals. Thirty minutes before sacrificing the mice, a 2.5% Evans blue solution in 1.5% methylcellulose (0.3 mL per animal) was intragastrically administered. After euthanasia, intestinal transit was measured as the distance from the pylorus to the most distal point of migration of the Evans blue dye. In addition, the length of the colon was measured.

### Tissue preparation and immunohistochemistry

Coronal slices of 40 μm were sectioned using a cryostat (CM3050, Leica Microsystems). Sections were incubated with 0.3% H_2_O_2_ for 30 min. Following blocking serum, sections were incubated overnight with rabbit anti-tyrosine hydroxylase (TH; Santa-Cruz Biotechnology) 1:1000 or with rat anti-dopamine transporter (DAT) 1:1000. Next day, sections were incubated with the appropriate biotinylated secondary antibody (Jackson ImmunoResearch) 1:200 for 2 h. The avidin-biotin method was used to amplify the signal (ABC Kit, Vector) and 3,3′-diaminobenzidine tetrachloride (DAB) was used as the chromogen.

The colons of the animals were embedded in paraffin. 15 μm sections were incubated with 0.3% H_2_O_2_ for 30 min, rehydrated and incubated with citrate buffer. Following serum block, sections were incubated overnight with the primary antibodies [rabbit anti-alpha-synuclein (1:1,000, millipore), rabbit anti-GFAP (1:1,000, Dako) or rabbit anti-CD3 (1:1,000, abcam)].

For alpha-synuclein and GFAP, slides were incubated with a fluorescent secondary antibody: Alexa®488 donkey anti-rabbit and mounted using Vectashield® mounting medium for fluorescence with DAPI (Vectro Laboratories). For CD3 staining, a biotinylated secondary antibody (1:200, Dako) was used. The avidin-biotin method was used to amplify the signal and DAB was used as chromogen. Sections were counterstained with Mayer's haematoxylin (Merck Millipore).

### Image analysis

For immunostained sections, digital images were captured with an Olympus BX50 microscope equipped with a Leica DFC 320 digital camera. To count TH-immunopositive cells, every fourth section of each mouse brain was stained. TH-immunopositive neurons were quantified stereologically on regular spaced sections. To measure DAT and alpha-synuclein expression the optical density in the area of interest was measured and corrected for non-specific background. Stereology was performed to quantify the number of CD3 positive cells in the colon on regular spaced sections. Analysis were performed by researchers that were blind to the treatment condition of the animal. Immunofluorescence images were made using a Keyence BZ-9000 microscope. The Corrected Total Fluorescence (CTF) was calculated with the formula: integrated density − (area × mean fluorescence of background reading).

### Statistical analysis

Experimental results are expressed as mean ± SEM. Differences between groups were statistically analyzed with a two-way ANOVA [analyzing significant effects of the treatments (rotenone vs. vehicle), diets (control diet, Diet1, and Diet2) and interactions (between diets and treatment)] followed by a Tukey's multiple comparison test. For the rotarod test and spatial memory test, data were analyzed with a general linear model repeated measure ANOVA with the within subject factor time and the between subject factors treatment and diet. Results were considered statistically significant when *p* < 0.05. Analyses were performed using SPSS 22.0.

## Results

### Dietary interventions have a restorative effect on rotenone-induced motor symptoms

**Figure 1:**
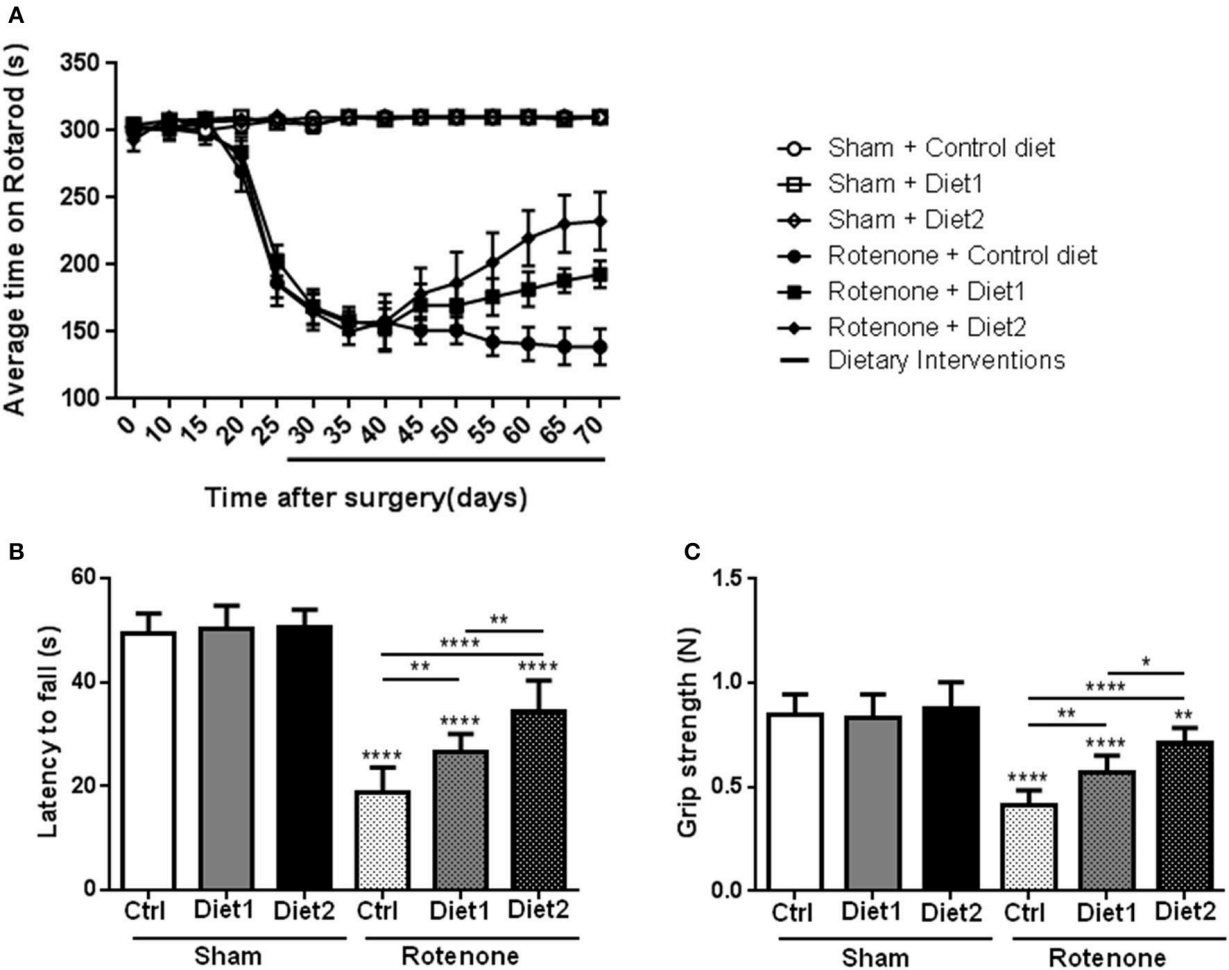
**Effects of the dietary interventions that were started after development of full motor dysfunction on motor symptoms;** **(A)** rotarod performance, **(B)** inverted screen test, and **(C)** forelimb grip strength test. Unilateral rotenone injection induced motor dysfunction and grip strength loss. Dietary interventions had beneficial effects on motor function and grip strength. The extended nutritional intervention (Diet2) was more effective than Diet1 for all the tests. Data are shown as mean ± SEM. ^*^p < 0.05, ^**^p < 0.01, ^****^p < 0.0001. (*n* = 10 per group).

To investigate whether dietary intake affected rotenone-induced motor dysfunction, the rotarod test was performed. The time spent on the rod was used for analysis of motor function. There was an overall effect of treatment (rotenone vs. vehicle) [*F*_(1, 44)_ = 492.62, *p* < 0.0001] and of diet [*F*_(2, 44)_ = 3.60, *p* < 0.05] on rotarod performance. Repeated measures showed an effect of time [*F*_(13, 572)_ = 50.58, *p* < 0.0001]. Rotenone-injected mice developed motor dysfunction over time compared to sham (interaction effect treatment x time *F*_(13, 572)_ = 59.25 *p* < 0.0001]. Furthermore, there was an interaction effect between diet and time [*F*_(26, 572)_ = 2.16, *p* < 0.01], between diet and treatment [*F*_(2, 44)_ = 3.55, *p* < 0.05] and between diet, treatment and time [*F*_(26, 572)_ = 2.15, *p* < 0.01]. Rotenone-injected mice showed a decrease in rotarod performance starting on day 25 after surgery compared to the sham-operated mice [*F*_(1, 54)_ = 226.41, *p* < 0.0001]. On day 55 after surgery there was a significant effect of the diets [*F*_(2, 53)_ = 3.30, *p* < 0.05] on rotarod performance that remained for the duration of the experiment [*F*_(2, 54)_ = 8.87, *p* < 0.0001 day 70]. *Post-hoc* analysis showed that rotenone-treated animals on Diet1 performed better on the rotarod compared to rotenone-treated animals on control diet starting from day 65 (*p* < 0.05). Rotenone-treated animals on Diet2 remained longer on the rod compared to rotenone treated animals on control diet starting from day 55 (*p* < 0.01). Moreover, Diet2 was significantly more effective in normalizing rotenone-induced motor-dysfunction compared to Diet1 (Figure 1A).

Muscle strength was measured with the inverted screen test 70 days after surgery, where the latency to fall was used for analysis. Significant differences were observed in the latency to fall of rotenone-injected mice compared to sham [*F*_(1, 53)_ = 417.5, *p* < 0.0001]. There was an overall effect of diet [*F*_(2, 53)_ = 17.41, *p* < 0.0001] and a diet by treatment interaction [*F*_(2, 53)_ = 13.10, *p* < 0.0001]. *Post-hoc* analysis revealed an increase in the latency to fall in the test in rotenone-treated mice on Diet1 (*p* < 0.01) and Diet2 (*p* < 0.0001) compared to rotenone-treated mice on Control diet. The extended nutritional intervention (Diet2) was more effective than Diet1 in reducing rotenone-induced muscle strength loss (*p* < 0.01; Figure 1B).

Similar results were found in the forelimb grip strength test, rotenone-treated animals showed a decrease in their forelimb grip strength compared to sham [*F*_(1, 53)_ = 130.1, *p* < 0.0001] 70 days after surgery. The diet had an overall effect on grip strength [*F*_(2, 53)_ = 14.7, *p* < 0.0001] and there was a diet by treatment interaction [*F*_(2, 53)_ = 9.3, *p* < 0.001]. More specifically, rotenone-injected mice fed the Diet1 and the Diet2 had more strength in the forelimbs compared to rotenone-injected mice on Control diet (*p* < 0.01 and *p* < 0.0001, respectively). Diet2 was again more effective than Diet1 in reducing rotenone-induced forelimb grip strength loss (*p* < 0.05; Figure 1C).

### Dietary interventions have no effects on rotenone-induced dopaminergic cell loss but Diet2 has restorative effects on dopamine transporter expression

**Figure 2:**
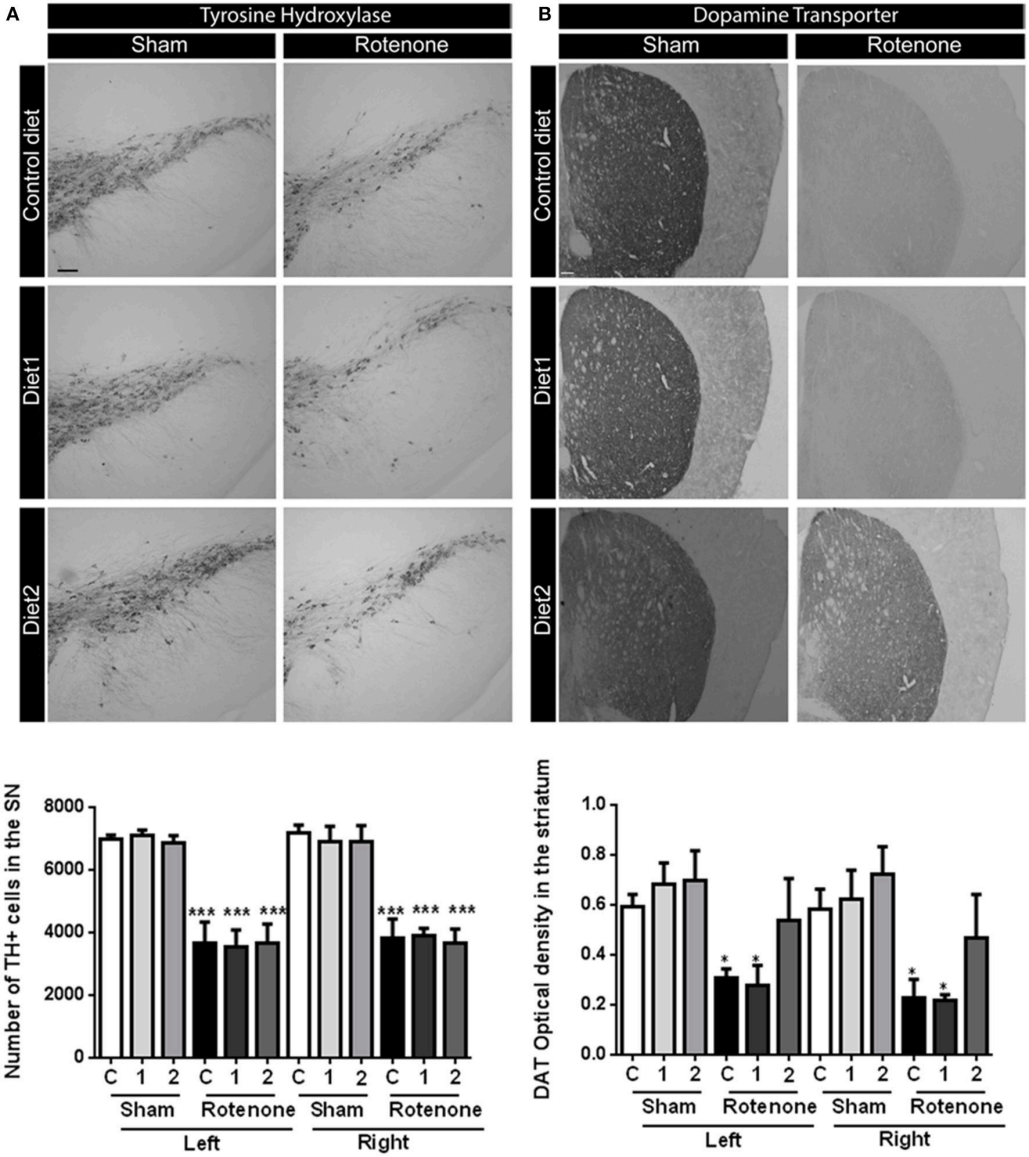
**Effects of dietary interventions that were started after development of full motor dysfunction on (A)** the number of dopaminergic cells indicated by the number of tyrosine hydroxylase (TH) immunoreactive cells in the substantia nigra and **(B)** dopamine transporter (DAT) expression in the striatum. Photographs show typical examples of TH and DAT immunostaining of substantia nigra and striatum, respectively, from all treatment groups. Rotenone injection decreased the number of dopaminergic cells in the substantia nigra and the level of DAT expression in the striatum. No differences were found in the number of TH positive cells and DAT expression between hemispheres after unilateral injection of rotenone. Dietary interventions had no effect on the number of TH positive cells but Diet2 increased DAT expression. Data are shown as mean ± SEM. ^*^p < 0.05, ^***^p < 0.001. (Scale bar: 200 μm applies to all panels). (*n* = 6 for TH immunostaining and *n* = 4 for DAT immunostaining).

To investigate the motor impairments associated neurodegeneration, we performed unbiased stereology to estimate the number of TH positive dopaminergic cells in the SN. Seventy days after surgery rotenone-treated mice showed a decrease in the number of TH positive cells compared to vehicle-injected mice [*F*_(1, 30)_ = 76.17, *p* < 0.001]. No differences were found in the total number of TH positive cells in the SN between the two brain hemispheres. There was no overall effect of diet [*F*_(2, 30)_ = 0.57, *p* = 0.57] and no significant interaction between treatment and diet [*F*_(2, 30)_ = 0.90, *p* = 0.42; Figure 2A).

Dopamine transporter (DAT) expression was measured in the striatum by measuring the optical density after DAT immunostaining. Rotenone caused a decrease in DAT expression in the striatum [*F*_(1, 36)_ = 26.49, *p* < 0.0001] 70 days after surgery. Unilateral-injection of rotenone caused a bilateral reduction of the DAT expression in the striatum; no differences were found in DAT immunoreactivity between the two brain hemispheres. There was an overall effect of the diets on DAT expression [*F*_(2, 36)_ = 3.44, *p* < 0.05; Figure 2B]. Diet2 increased DAT expression as compared to Control diet in rotenone-treated mice, in the absence of a significant treatment by diet interaction.

### Diet 2 has restorative effects on rotenone-induced spatial recognition impairment

**Figure 3:**
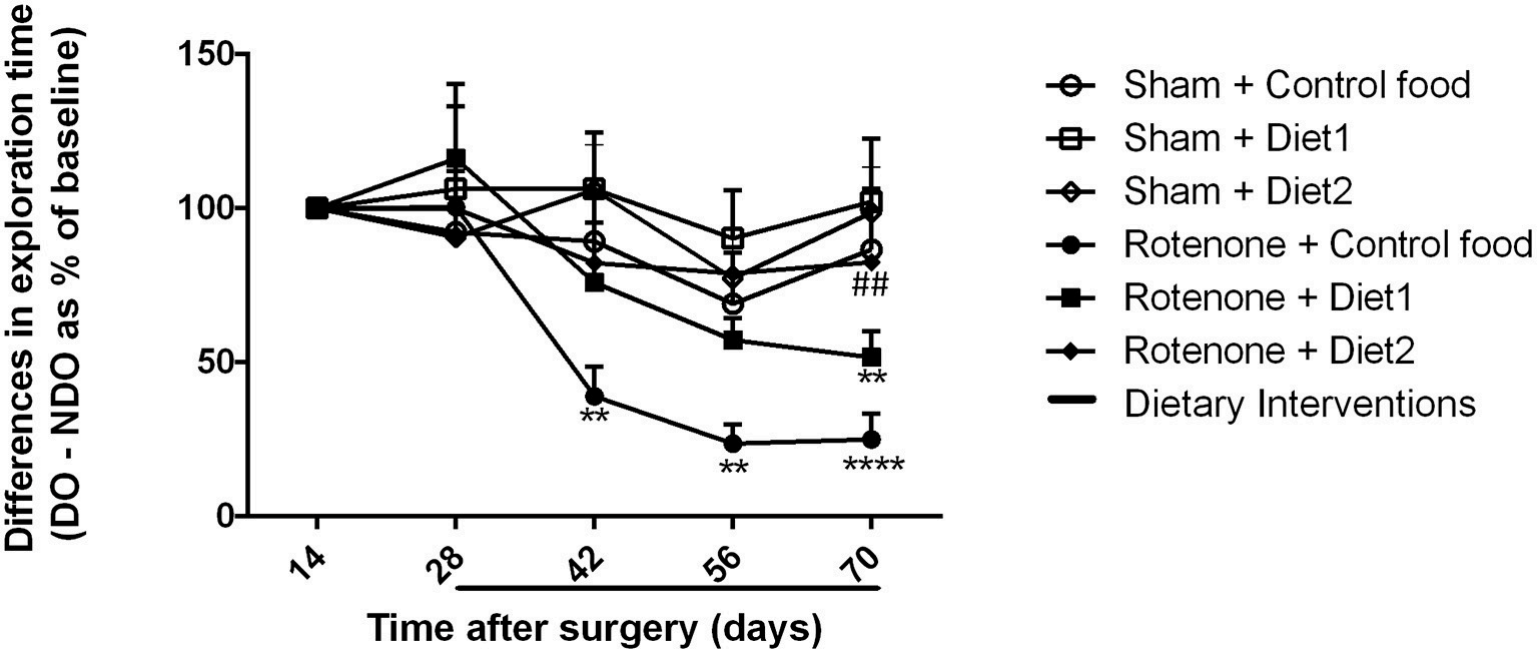
**Effects of dietary interventions that were started after development of full motor dysfunction on spatial object recognition test**. Sham-operated animals selectively re-explored the displace object (DO) as compared to the non-displaced object (NDO) throughout the experiment. Rotenone decreased animals' ability to react to a spatial novelty from day 42 after surgery onwards. On day 70 after surgery, rotenone-injected animals on Diet2 showed better spatial discrimination abilities compared to rotenone-injected animals on control diet. Data are shown as mean ± SEM. ^**^p < 0.01, ^****^p < 0.0001 compared to Sham + Control food. ^*##*^*p* < 0.01 compared to Rotenone + Control food (*n* = 10 per group).

Sham-operated animals selectively re-explored the displace object (DO) as compared to the non-displaced object (NDO) during the whole experiment, demonstrating that they were able to selectively react to the spatial change. Rotenone treatment negatively affected the rodents' ability to react to a spatial novelty [*F*_(1, 54)_ = 4.701, *p* < 0.05]. Repeated measures showed an effect of time [*F*_(4, 216)_ = 11.06, *p* < 0.0001] and an effect of rotenone over time [*F*_(4, 216)_ = 5.82, *p* < 0.0001]. Spatial recognition was affected by rotenone from day 42 after surgery onwards [*F*_(1, 54)_ = 7.10, *p* < 0.05] and by the diet on day 56 after surgery [day 28 after dietary interventions started; *F*_(2, 54)_ = 3.52, *p* < 0.05]. Post-hoc comparisons showed no differences in spatial discrimination abilities between rotenone-treated mice on Control diet and on Diet1. However, rotenone-injected mice on Diet2 had a better spatial recognition than rotenone-injected animals on Control diet (*p* < 0.05) on day 70 (Figure 3). No significant differences were found in the total activity in the open field between experimental groups.

### Diet 2 has a restorative effect on rotenone-induced gastrointestinal dysfunction

**Figure 4:**
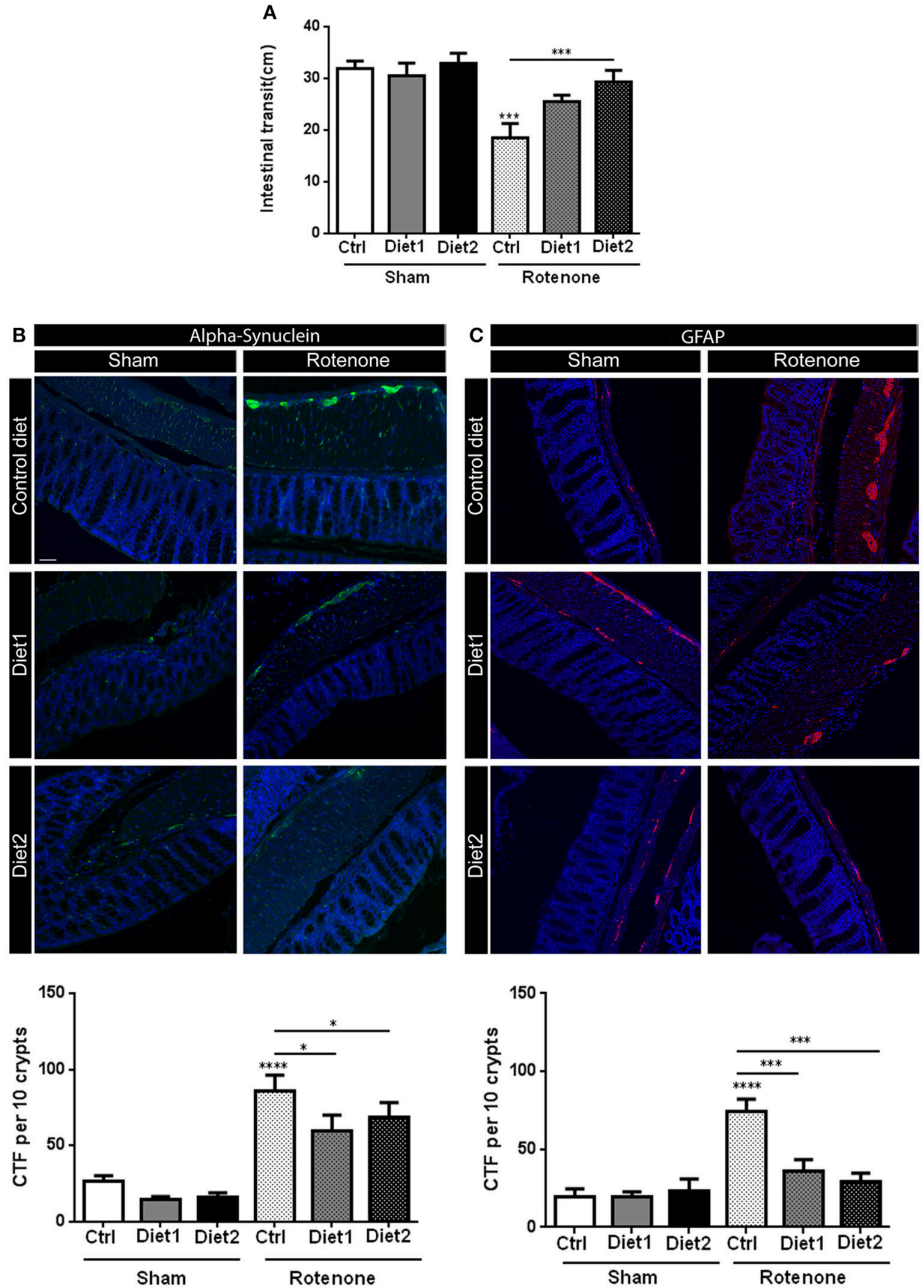
**Effects of dietary interventions that were started after development of full motor dysfunction on: (A)** intestinal transit indicated by the total distance traveled by the Evans blue dye in the GI tract 30 min after its injection by oral gavage, **(B)** alpha-synuclein expression in the colon and **(C)** on enteric glial cells expression in the colon as indicated by the glial fibrillary acidic protein (GFAP). Rotenone injection reduced intestinal transit time and increased alpha-synuclein (shown in green) and GFAP (shown in red) expression in the myenteric and submucosal plexus of the colon. Dietary interventions improved rotenone-induced delayed intestinal transit and reduced rotenone-induced alpha-synuclein and GFAP overexpression in the colon. The beneficial effect of Diet2 on intestinal transit after rotenone exposure was more pronounced that the effect of Diet1. The Corrected Total Fluorescence (CTF) was calculated with the formula: integrated density-(area × mean fluorescence of background reading). Data are shown as mean ± SEM. ^*^p < 0.05, ^***^p < 0.001, ^****^p < 0.0001. (Scale bar: 50 μm applies to all panels) (*n* = 9 or 10 per group).

Rotenone injection in the striatum negatively affected intestinal transit, i.e., decreased the distance traveled by the Evans Blue dye in the GI-tract, as compared to vehicle-injected mice [*F*_(1, 51)_ = 18.81, *p* < 0.0001]. There was an overall effect of diet on intestinal transit [*F*_(2, 51)_ = 4.19, *p* < 0.05] and an interaction effect between treatment and diet [*F*_(2, 51)_ = 3.33, *p* < 0.05]. The beneficial effect of Diet2 on intestinal transit after rotenone exposure was more pronounced (*p* < 0.001; Figure 4A).

### Dietary interventions have a restorative effect on rotenone-induced alpha-synuclein accumulation in the colon

Rotenone administration in the striatum increased alpha-synuclein accumulation in the enteric plexus of the colon compared to vehicle exposed mice [*F*_(1, 51)_ = 72.14, *p* < 0.0001]. There was an effect of diet on alpha-synuclein accumulation in the colon [*F*_(2, 51)_ = 3.56, *p* < 0.05]. *Post-hoc* analysis revealed a reduction in alpha-synuclein levels for rotenone-injected mice on Diet1 and Diet2 compared to rotenone-injected mice on Control diet (both *p* < 0.05). No differences were found in alpha-synuclein levels for rotenone-animals on Diet1 compared to rotenone-treated animals on Diet2 (Figure 4B).

### Dietary interventions have a restorative effect on rotenone-induced inflammation in the gut

To measure reactive enteric glial cells in the enteric nervous system (ENS) of the colon, GFAP (glial fibrillary acidic protein) expression was quantified after immunostaining. Rotenone exposure in the brain increased GFAP expression in the colon [*F*_(1, 54)_ = 23.94, *p* < 0.0001]. There was also an effect of diet [*F*_(2, 54)_ = 6.36, *p* < 0.01] and an interaction effect between diet and treatment [*F*_(2, 54)_ = 7.83, *p* < 0.01]. *Post-hoc* analysis showed that GFAP expression was normalized in rotenone-injected mice on Diet1 or Diet2 compared to rotenone-treated animals on Control diet (both *p* < 0.001; Figure 4C).

**Figure 5:**
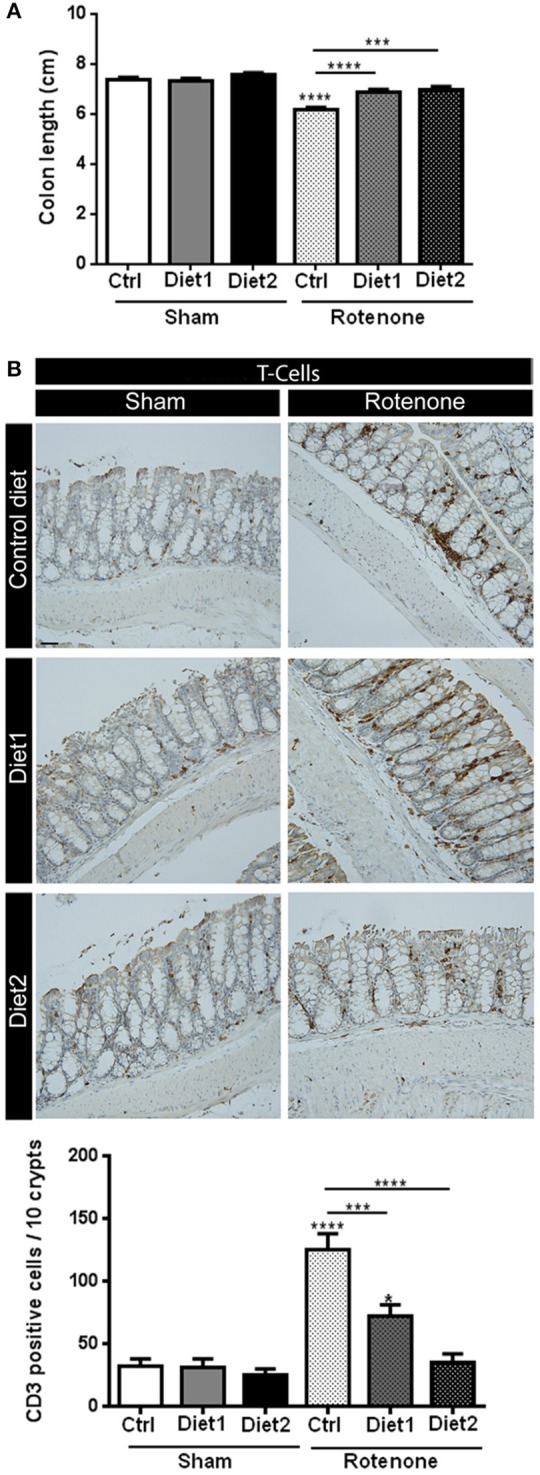
**Effects of dietary interventions that were started after development of full motor dysfunction on (A)** colon length and **(B)** the number of T-cells in the colon. Photographs show typical examples of T-cell immunostaining in the colon from all treatment groups. Rotenone reduced the length of the colon and increased the number of T-cells (shown in brown). Both dietary interventions increased rotenone-induced reduction of the colon and reduced T-cell infiltration. Diet2 was more effective in normalizing rotenone induced T-cell infiltration than Diet1. Data are shown as mean ± SEM. ^*^p < 0.05, ^***^p < 0.001, ^****^p < 0.0001. (Scale bar: 50 μm applies to all panels). (*n* = 10 per group for colon length and *n* = 7 for T-cell infiltration 10).

The length of the colon of all the animals was measured as a gross indicator of inflammation. There was an overall effect of the treatment [rotenone vs. vehicle; *F*_(1, 54)_ = 72.25, *p* < 0.0001] as well as an effect of diet [*F*_(2, 54)_ = 10.42, *p* < 0.001] and an interaction effect between treatment and diet [*F*_(2, 54)_ = 7.32, *p* < 0.01]. More specifically, rotenone-treated animals on Diet1 and Diet2 revealed a smaller decrease in colon length compared to the ones on Control diet (*p* < 0.0001 and *p* < 0.001, respectively). No differences in colon length were found between rotenone-treated mice on Diet1 and rotenone-treated mice on Diet2 (Figure 5A).

The number of T-cells in the colon was increased after rotenone injection in the brain [*F*_(1, 36)_ = 51.41, *p* < 0.0001]. There was an effect of diet [*F*_(2, 36)_ = 17.48, *p* < 0.0001] and an interaction effect between treatment and diet [*F*_(2, 36)_ = 12.80, *p* < 0.0001]. *Post-hoc* analysis revealed that rotenone-injected mice on Diet1 and Diet2 had less infiltration of T-cells in the colon compared to rotenone-injected mice on Control diet (*p* < 0.001 and *p* < 0.0001, respectively). Diet2 was more effective than Diet1 in reducing rotenone-induced T-cell infiltration (*p* < 0.05; Figure 5B).

## Discussion

In the present study we replicated previous finding (Perez-Pardo et al., 2017) by showing that unilateral rotenone injection in the striatum caused a disturbed motor function, bilateral dopaminergic cell loss in the SN, delayed intestinal transit, alpha-synuclein accumulation in the ENS, and colonic inflammation. In addition, we demonstrated that intrastriatal rotenone injection caused grip strength loss, spatial recognition deficits, and an increase in the amount of reactive enteric glial cells in the colon. These new findings broaden the range of motor and non-motor symptoms observed and increase the relevance of the model for PD in patients where cognitive symptoms (Boller et al., 1984; Giraudo et al., 1997) and increased inflammation markers in colonic biopsies are reported (Clairembault et al., 2014). Furthermore, in our mouse model the spatial recognition deficits developed later than the motor dysfunction, similar to PD patients where cognitive symptoms tend to occur later in the course of the disease. Therefore, the intrastriatal rotenone model in mice is a consistent model of PD that recapitulates key features of the pathology and symptomatology observed in humans. Whether other non-motor symptoms, in addition to cognitive and gastrointestinal dysfunction, are also induced by the model remains to be determined.

In the present study we also extended our recent observations regarding the effects of specific dietary interventions on a broad range of motor and non-motor symptoms in this mouse model of PD. It was previously shown that uridine and DHA were able to reduce circling behavior in the 6-OHDA animal model (Cansev et al., 2008) and we showed that uridine and DHA prevented the induction of motor deficits as well as the development of GI dysfunctions in rotenone models of PD (Perez-Pardo et al., 2017). Here, we show that the same diet (Diet1) given after the occurrence of motor problems, was able to reduce motor dysfunction, grip strength loss, cognitive deficits, delayed intestinal transit, colonic inflammation, and alpha-synuclein accumulation in the ENS. This is the first study demonstrating a clear therapeutic effect of specific dietary interventions in a mouse model for PD.

Dietary interventions were started after the motor symptoms had clearly developed. As a consequence, we see no effects of the diets on the number of dopaminergic cells in the SN, indicating that the diets did not reduce the loss of dopaminergic cells, i.e., they did not interfere with rotenone toxicity. The diets helped to improve the functioning of the remaining dopaminergic neurons, demonstrating that they have neurorestorative properties and therefore may have disease-modifying potential.

There are several relevant mechanisms that may have contributed to the restoration of functions observed in the present study. A series of animal studies have shown that combined intake of phospholipid precursors including uridine and omega-3 fatty acids such as DHA, can increase brain phospholipid levels, synaptic protein levels, neurite outgrowth, dendritic spine formation, and dopaminergic neurotransmission (Wang et al., 2005; Sakamoto et al., 2007; Wurtman et al., 2009, 2010). Similarly to previous observations in the 6-OHDA rat model of PD, where uridine plus DHA restored nigrostriatal markers and ameliorated biochemical defects (Cansev et al., 2008), it is likely that a diet-induced enhancement of functional connectivity may underlie the presently observed recovery from behavioral deficits.

In addition to replicating the beneficial effects of Diet1 in the striatal rotenone model, we show that the extended nutritional intervention (Diet2) containing both precursors and other nutrients that increase phospholipid synthesis as well as prebiotic fibers was more effective in normalizing rotenone-induced motor and non-motor abnormalities. Although the present study was not designed to test the contribution of all individual nutrients, there are several factors that may have contributed to this enhanced effectiveness.

First, Diet2 contained specific prebiotic fibers such as fructo-oligosaccharides and galacto-oligosaccharides that have previously been shown to have beneficial effects on immune function (van Hoffen et al., 2009; de Kivit et al., 2011), bowel motility, and constipation (Scholtens et al., 2014; Meksawan et al., 2016), by altering the microbiota composition and their metabolic activity. Changes in microbiota composition might also alter the enteric immune and nervous system and subsequently the central nervous system (Maslowski and Mackay, 2011; Clemente et al., 2012; Cryan and Dinan, 2015). In addition, the prebiotic fibers have microbiota-independent immunomodulatory properties (de Kivit et al., 2013). Thus, adding prebiotic fibers in Diet2 may have contributed to its better effects on intestinal transit and colonic inflammation compared to Diet1, and possibly also to the beneficial effects on motor and cognitive functioning.

Second, Diet2 contained a broader combination of nutrients that has been shown to act synergistically to increase the synthesis of synaptic membranes (van Wijk et al., 2014). On the one hand it contained a full set of precursors for membrane synthesis (i.e., DHA, EPA, uridine, choline, and phospholipids) that act by enhancing the substrate-saturation of the enzymes that catalyze the rate-limiting steps of phospholipid synthesis. On the other hand it also contained B-vitamins, vitamin C, vitamin E, selenium, and phospholipids that act as cofactors by increasing the availability of membrane precursors or by directly affecting the neuronal membrane or membrane synthesis (van Wijk et al., 2014, 2016). Next to synergies between individual precursors (Wurtman et al., 2009, 2010), the added value of the cofactors has for instance been reported on the activation of G-protein-coupled receptors (Savelkoul et al., 2012). In several *in vivo* experiments, the diet has repeatedly been shown to be more effective than supplementation of single nutrients or incomplete nutritional combinations (Broersen et al., 2013; Zerbi et al., 2014; Janickova et al., 2015). Also in the present study Diet2 was more effective than the combination of uridine plus DHA (Diet1) on all functional and behavioral parameters. In addition, only intervention with this extended diet resulted in a restoration of the expression of DAT in the striatum, which might be one of the mechanisms by which the diet enhances dopaminergic transmission in the remaining nigrostriatal neurons resulting in a better functional output. Together these data suggest that especially the combined intake of these nutrients, rather than the use of single component supplements, could be beneficial to maintain brain structure and function.

The present results regarding the effectiveness of the extended dietary intervention (Diet2) in restoring functional connectivity in the rotenone model of PD are in line with previous observations in other models of either ongoing neurodegeneration (Jansen et al., 2013; Wiesmann et al., 2016) or acute neurotrauma (Pallier et al., 2015; Wiesmann et al., 2017). In these experiments Diet2 improved both neuronal connectivity and behavioral output, suggesting a broad applicability of this nutritional technology. This, together with positive results regarding improved brain connectivity and functioning obtained in patients with AD (Scheltens et al., 2012; de Waal et al., 2014) and preliminary findings in patients with frontotemporal dementia (Pardini et al., 2015), as well as a favorable safety profile of the intervention (Cummings et al., 2017) warrant further clinical testing in other patient populations (Cummings, 2012), including PD.

In summary, our study demonstrated clear therapeutic effects of specific dietary interventions in a mouse model of PD given after disease induction. The extended nutritional intervention (Diet2) was more effective than supplementation of only uridine and DHA (Diet1) in normalizing rotenone-induced motor and non-motor symptoms and PD-like pathologies in brain and gut. Our results suggest that this nutritional intervention might confer clinical benefits to patients suffering from PD.

## Author contributions

PP, ED, LB, NV, AA, JG, and AK conceived and designed the experiment. PP performed the animal experiments with ED's assistance. Immunostainings were performed by PP and ED. Statistical analysis were designed by PP and LB and performed by PP. PP, ED, LB, NV, and AK interpreted the statistical outcome. PP wrote the first draft of the manuscript, ED, LB, NV, AA, JG, and AK reviewed and critiqued the manuscript. PP performed the figures layout. All authors were responsible for the decision to submit the manuscript for publication.

## Funding

This study is part of the Utrecht University “Focus en Massa program” and financially supported by Nutricia Research.

### Conflict of interest statement

LB, NV, AA, and JG are employees of Nutricia Research, Utrecht, Netherlands. The other authors declare that the research was conducted in the absence of any commercial or financial relationships that could be construed as a potential conflict of interest.
